# Axillary papules: an uncommon location of lichen nitidus^☆^^☆☆^

**DOI:** 10.1016/j.abd.2020.04.015

**Published:** 2021-03-16

**Authors:** Walter Belda, Paulo Ricardo Criado, Nilton Gioia Di Chiacchio

**Affiliations:** a Faculdade de Medicina, Universidade de São Paulo, São Paulo, SP, Brasil; b Faculdade de Medicina do ABC, São Paulo, SP, Brazil; c Hospital do Servidor Público Municipal de São Paulo, São Paulo, SP, Brazil

Dear Editor,

Lichen nitidus is a relatively rare, chronic, papulosquamous cutaneous disease that is characterized by multiple flesh-coloured shiny, dome-shaped papules, with sizes from 1 to 2 mm, often referred as pinhead-like papules.^1^ The crop of the lesions often is asymptomatic; moreover, it sometimes may associate with pruritus.^1^ This uncommon condition was described for the first time by Pinkus in 1901.^2^ The skin is the primary site involved but the mucous membranes and nails also might be affected.^3^

No racial or sex predilection is reported, although the majority of cases appear to arise in children and young adults.^1^, ^4^ There are located and generalized forms of lichen nitidus, sometimes described under clinical variants: familiar, actinic, confluent, vesicular, hemorrhagic, palmo-plantaris, mucous, spinulosus and follicularis, keratodermic, perforating or linear.^2^, ^5^ The lesions are located preferentially on the flexor surface of the arms, wrists, on the abdomen and genitalia, though they can become disseminated.^5^ We are adding to the indexed literature the second case of lichen nitidus exclusively located on both axillae.

The patient is a 26-year-old Caucasian man who was seen for evaluation of asymptomatic lesions on the both axillae; the lesions had been present more than 4-years and showed insidious emergence. He denied previous treatment on the lesions or any medication intake preceding the crop of the lesions. On his dermatological exam, discrete or grouped skin-colored, shiny, firm, monomorphic round, and dome-topped papules of 1-to 3-mm in diameter were observed on both axillae (Fig. 1).

**Figure 1:**
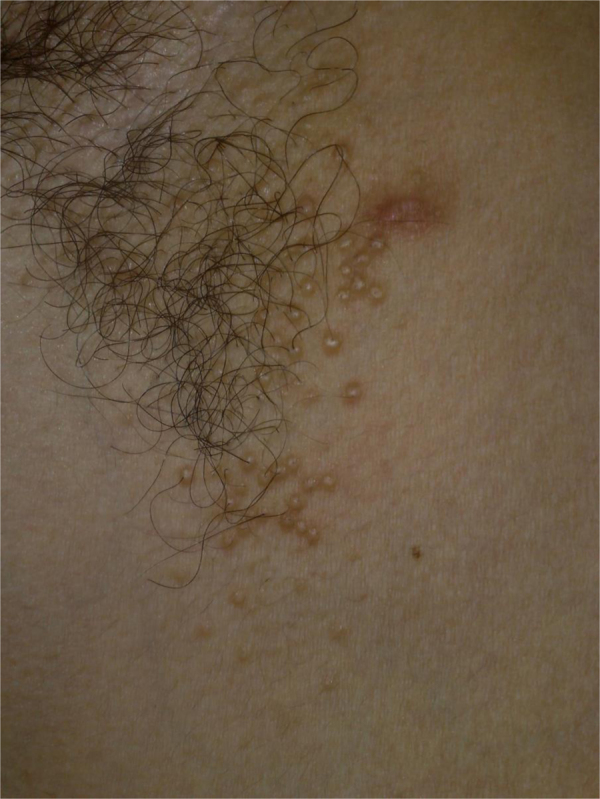
Right axilla presenting discrete and grouped skin-colored, shiny, firm, monomorphic round, and dome-topped papules of 1-to 3-mm.

A skin biopsy was performed from these lesions, and that displayed a lymphohistiocytic infiltrate in a broadened dermal papilla, with a descending growth of the rete ridges surrounding the dermal inflammatory infiltrate in a “ball-and-claw” manner (Figure 2, Figure 3). The overlying epidermis was noted to be unremarkable, and there was no evidence of spongiosis or exocytosis.

**Figure 2:**
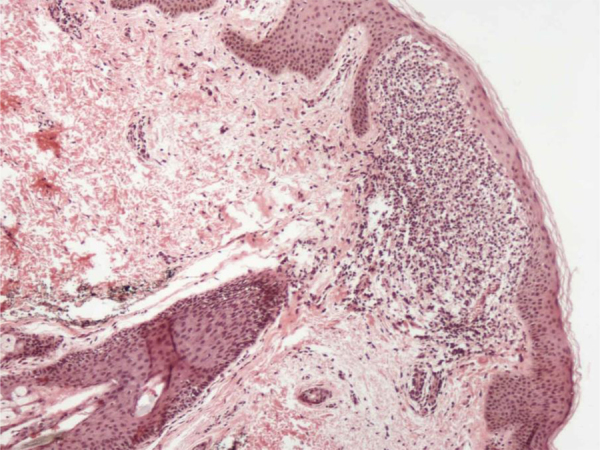
Infiltrate in a broadened dermal papilla, with a descending growth of the rete ridges surrounding the dermal inflammatory infiltrate in a “ball-and-claw” manner, (Hematoxylin & eosin, ×40).

**Figure 3:**
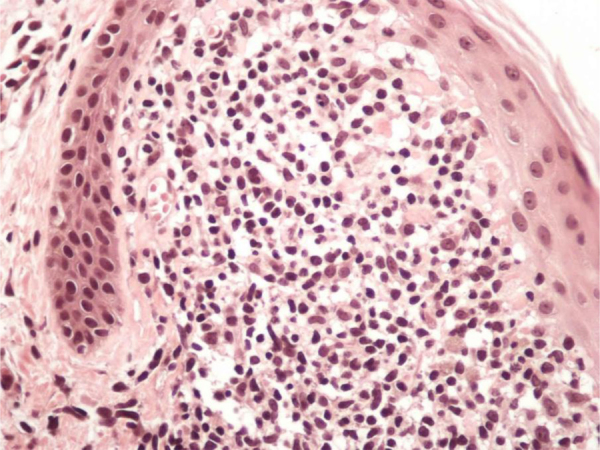
Higher magnification showing the lymphohistiocytic infiltrate in a broadened dermal papila, (Hematoxylin & eosin, ×200).

The patient was treated with the combination of dexchlorpheniramine 2 mg and betamethasone 0.25 mg t.i.d per os for 10-days, and after thatt he was virtually clear of lesions.

There is only one report of lichen nitidus on axillae.^3^ Our patient displayed lesions only in this area, emphasizing the peculiar aspect of our report. Once considered as a tuberculoid reaction, lichen nitidus is currently regarded as a disorder of unknown origin. The differential diagnosis includes lichen planus, psoriasis, verruca plana and keratosis pilaris.^1^, ^4^ Rare cases of lichen nitidus associated with atopic dermatitis, Crohn disease, Down´s syndrome and juvenile chronic arthritis have been reported.^1^, ^3^ The first clinical hypothesis in this case was Fox-Fordyce disease due the presence of popular lesions on the axillae. The final diagnosis was established on histopathological basis.

Due to the rare clinical presentation of lichen nitidus exclusively located on the both axillae, the clinicians must alert to the necessity of performing cutaneous biopsy to confirm this diagnosis. The histological study of the biopsy showed characteristic findings of lichen nitidus, including lymphohistiocytic infiltrate in an expanded dermal papilla with thinning of overlying epidermis and downward extension of the rete ridges at the lateral margin of the infiltrate, producing a typical "claw clutching a ball" picture.^1^, ^5^

In two-thirds of patients the lesions resolve spontaneously after months to 1 year. Topical glucocorticoids can be useful in localized forms.^1^

In this case, the patient was successfully treated with the combination of dexchlorpheniramine 2 mg and betamethasone 0.25 mg t.i.d per os for 10-days.

The authors would like to highlight the rarity of the presentation of this case and the importance of considering the lichen nitidus as a differential diagnosis of papular lesions in the axillae, itchy or not.

## Financial support

None declared.

## Authors’ contributions

Walter Belda Junior: Statistical analysis; approval of the final version of the manuscript; design and planning of the study; drafting and editing of the manuscript; collection, analysis, and interpretation of data; effective participation in research orientation; intellectual participation in the propaedeutic and/or therapeutic conduct of the studied cases; critical review of the literature; critical review of the manuscript.

Paulo Ricardo Criado: Statistical analysis; approval of the final version of the manuscript; design and planning of the study; drafting and editing of the manuscript; collection, analysis, and interpretation of data; effective participation in research orientation; intellectual participation in the propaedeutic and/or therapeutic conduct of the studied cases; critical review of the literature; critical review of the manuscript.

Nilton Gioia Di Chiacchio: Statistical analysis; approval of the final version of the manuscript; design and planning of the study; drafting and editing of the manuscript; collection, analysis, and interpretation of data; effective participation in research orientation; intellectual participation in the propaedeutic and/or therapeutic conduct of the studied cases; critical review of the literature; critical review of the manuscript.

## Conflicts of interest

None declared.
